# Probiotic *Lactobacillus plantarum* IS 10506 supplementation increase SCFA of women with functional constipation

**Published:** 2019-10

**Authors:** Pratiwi Dyah Kusumo, Hasan Maulahela, Amanda Pitarini Utari, Ingrid S. Surono, Amin Soebandrio, Murdani Abdullah

**Affiliations:** 1 Biomedic Doctoral Programme, School of Medicine, Universitas Indonesia, Jakarta, Indonesia; 2 Department of Biomedic, School of Medicine, Universitas Kristen Indonesia, Jakarta, Indonesia; 3 Department of Gastroenterology, School of Medicine, Universitas Indonesia, Jakarta, Indonesia; 4 Department of Food Technology, Faculty of Engineering, Bina Nusantara University, Jakarta, Indonesia; 5 Eijkman Biology Molecular Institute, Jakarta, Indonesia; 6 Department of Microbiology, School of Medicine, Universitas Indonesia, Jakarta, Indonesia

**Keywords:** Short chain fatty acid, *Lactobacillus plantarum*, Functional constipation, Probiotic

## Abstract

**Background and Objectives:**

Gut microbiota influences our health via multiple mechanisms. Microbiota produced Short Chain Fatty Acid (SCFA) as an energy to maintain gut ecosystem and physiology. Dysbiosis is correlated with SCFA imbalance which in turn resulted in physiological abnormalities in the intestine, such as functional constipation.

**Materials and Methods:**

Randomized Double-Blind Controlled Trial (RCT) was conducted on women with functional constipation (n=37) in the community of Jakarta and profile of SCFA was assessed by using GC-MS from the stool after 21 days supplementation of fermented milk (placebo and probiotic).

**Results:**

Probiotic supplementation significantly influenced acetate titer (p=0,032) marginally significant for propionate and butyrate (p=0.063 and p=0.068, respectively) and the respondent with increasing SCFA’s metabolite are higher in probiotic group compared to the respondents in placebo group. Acetate is the highest SCFA titer found in faeces samples of women with functional constipation.

**Conclusion:**

Probiotic *Lactobacillus plantarum* IS 10506 supplementation influenced all the SCFA parameter (acetate, propionate and butyrate).

## INTRODUCTION

Functional constipation is caused by a motility disorder without structural abnormalities. Indonesian Gastroenterology Administration (GI) observed a tendency of increasing incidence of constipation, and constipation patients tended to carry out self-medication, by buying over the counter (OTC) that generally inappropriate, hence, we explore the potency of probiotic function toward the improvement measurement symptom of functional constipation using PAC-SYM^©^ questionnaire, which is expected to facilitate in balancing microbiota composition and suppress dysbiosis. The research on functional constipation was conducted on the risk factors (gender, social factors, Body Mass Index (BMI), activity, diet and age) but has not been based on the scientific evidences of the causal factors and underlying mechanisms (1). Among the risk factors, women are the dominant sex factor, therefore the subjects were restricted to women. There are three factors involve in mechanism of functional constipation due to functional disorders of digestive tract: host factors (immunity), luminal factors and environmental factors. Luminal factors (one of which is dysbiosis) are assumed to be strong association with other factors and considered to be the foundation of several diseases. The imbalance of microbiota in the digestive tract may affect the metabolites such as Short Chain Fatty Acid (SCFA) as a result of carbohydrates fermentation and proteins in the lower digestive tract (2, 3).

SCFAs refer to free fatty acids containing fewer than 6 carbons with short aliphatic carbon-chains. Formic acid (C1), acetic acid (C2), propionic acid (C3), butyric acid (C4), and valeric acid (C5) are the main products of carbohydrate metabolism by intestinal microbiota. Lactate, although not belong to SCFA, is also produced by several microbiota such as lactic acid bacteria, bifidobacteria and proteobacteria. *Eubacterium hallii* can convert lactate to a different SCFA under the normal condition and mostly abundant in colon. SCFA can be oxidized to provide energy and have also been shown to affect the immune system, colonic function, cholesterol metabolism, satiety and oxidative stress. SCFAs, particularly C4, are utilized as major energy source for colonic epithelial cells and regulate their gene expression, proliferation, differentiation, and apoptosis. SCFAs promote the production of mucin and gastrointestinal antimicrobial peptide (e.g.LL-37) molecules important for gut barrier function (4–6). SCFA has a role in physiological processes in the colon and will affect the defecation process. We measured SCFA’s titer (butyrate, propionate and acetate) as an improvement parameter of women with functional constipation after 21 days supplementation of fermented milk containing probiotic *Lactobacillus plantarum* IS 10506 isolated from Dadih, West Sumatra (7).

## MATERIALS AND METHODS

### Study Design

This study was a Randomized Double-Blind Controlled Trial (RCT) study conducted for 3 weeks, with double-blind procedures and parallel groups applied. All subjects were randomly divided into 2 groups, Placebo group (21 respondents) and Probiotic group (16 respondents), and were informed from the start that there were subjects who received placebo (lactic acid dextrose milk) and probiotic (fermented milk containing *Lactobacillus plantarum* IS 10506, 10^10^ CFU/mL).

### Study location

This research was conducted within the scope of the sampling Jakarta area in community (Petamburan Health Center, Jakarta). SCFA’s analysis was conducted in Parasitology Department Laboratory Faculty of Medicine Universitas Indonesia and Regional Health Laboratory of Jakarta (Labkesda).

### Sampling

Subjects were obtained from populations living in the community living near Petamburan district in the capital city of Jakarta, Indonesia. There were 37 women, 18–60 years old, with functional constipation symptoms selected using inclusion and exclusion criteria based on Rome IV by competent medical personnel, and supplemented with 120 ml fermented milk for 21 days. The adherence was monitored to check consumption of fermented milk one bottle/day during supplementation period, and the fermented milk was kept in a refrigerator (minimum 8°C).

### Stool collection and extraction

Faecal samples were collected in a pot, immediately cooled in a cool box, and then stored in −20°C freezer. There were three times of sample collections (Baseline-Interval-Endline). Faeces aliquots of 1 g of dry weight each, were transferred into 25 mL plastic vial, added with 3 ml ethyl acetate and 3 ml formic acid, homogenized with vortex and then centrifuged for 10 minutes at 3000 g. The supernatant (organic phases) was separated and transferred into 15 mL plastic vial that had been added with Na_2_SO_4_ anhidrate. The samples were stored at −20°C until analysis with gas chromatography–mass spectrometry (GC-MS) in Labkesda.

### GC-MS analysis

Data were analyzed using Agilent Technology 6890 Gas Chromatogarphs with auto samplers and 5973 Mass Selective Detection and Chemotation Data System. Samples (5 μl) were directly injected into the gas chromatograph equipped with an HP-Innowax capillary column (30-m length by 0.25-mm internal diameter, with a 0.25 μm film thickness; Agilent) using He as gas carrier and a constant flow rate of 0.8 ml/min. The temperature of the injector was kept at 230°C, and the split ratio was 8:1. Chromatographic conditions were as follows: initial oven temperature of 80°C, 8°C/min up to 220°C, 12 min at 220°C, and a ramp of 20°C/min up to 230°C to clean the column. In the MS detector, the electron impact energy was set at 70 eV. The data collected in method file (FFAMS).

### Statistical analysis

The data experiment was analyzed using SPSS version 22.0 (IBM SPSS Statistics for windows version 22.0 Armonk, NY; IBM Corp). Normality distributed data test using Shapiro-wiro, non parametric test MannWhitney and Chi-Square test was used to evaluate the effect of probiotic supplementation on SCFA titer. Spearman Rho test was performed to evaluate the correlation between variation of treatment and SCFA titer. The level of P value < 0.05 was considered as significant.

## RESULTS

### Respondent profile

Women respondent was 18–60 years and had a body mass index (BMI) of 14.68 to 34.95 kg/m^2^ inclusive (Table 1). Most of respondents in Petamburan were house wives, and only some of them were part time worker. Thirty subjects were included in this study, divided into 21 respondents in placebo group and 16 respondents in probiotic group. In general human physiology processes are influenced by age and BMI as well as metabolism in the digestive tract, one of which is SCFA production. We analyzed the correlation between age and BMI that will influenced the SCFA metabolites (Table 2). We found that age had weak negative correlation with delta acetate and propionate (p=−0.023 and p=-0.242 respectively) and weak positive correlation with delta butyrate (p=0.085). Correlation between Post BMI and delta SCFA also weak correlation, acetate, propionate and butyrate (p=0.127, p=−0.040 and p=0.054 respectively) according to Spearman-Rho test correlation.

**Table 1:** Participants socio-demography and anthropometric data

Characteristic*	**Placebo (n 15)**	**Probiotic (n 15)**
Age (year)	45.52 ± 10.71	34.50 ± 12.33
Height (meter)	1.55 ± 0.07	1.60 ± 0.05
Baseline Weight (kg)	61.81 ± 13.00	64.19 ± 13.97
Baseline BMI (kg/m^2^)	25.41 ± 5.00	25.05 ± 5.40
Endline Weight (kg)	62.43 ± 12.00	65.00 ± 14.14
Endline BMI (kg/m^2^)	25.67 ± 4.56	25.36 ± 5.39

BMI: Body Mass Index.

* value are expressed as mean ± Standard deviation

**Table 2:** Profile SCFA (Baseline, Interval, Endline)

**Characteristic SCFA titer (mM)**	**Placebo (*n* 21)**	**Probiotic (*n* 15)**
Baseline-Acetate	52.17 ± 45.38	53.93 ± 32.99
Interval-Acetate	80.17 ± 59.19	66.18 ± 51.20
Endline-Acetate	41.38 ± 38.23	81.51 ± 61.67
Baseline-Propionate	24.94 ± 23.50	21.85 ± 14.42
Interval-Propionate	38.06 ± 46.91	34.97 ± 29.76
Endline-Propionate	17.82 ± 21.29	41.60 ± 37.13
Baseline-Butyrate	43.29 ± 26.25	40.26 ± 20.62
Interval-Butyrate	39.04 ± 25.89	43.11 ± 25.61
Endline Acetate	41.38 ± 38.23	81.51 ± 61.67

### SCFA profile

We performed three measurements of SCFA metabolites (baseline, interval and endline), to find out how the changes that occurred during the supplementation process and the effect of probiotic supplementation on these changes.

Probiotic *Lactobacillus plantarum* IS 10506 supplementation has an effect on the percentage of delta (endline-baseline/baseline) SCFA after 21 days (Table 2). According to the Mann-Whitney test, probiotic effect to the percentage changes of acetate, propionate and butyrate profile (p=0.032, p=0.063, p=0.068 respectively). Probiotic *Lactobacillus plantarum* IS 10506 as luminal factor assumed strongly associated with SCFA metabolite. Total respondents with increasing SCFA concentration tend to be different in placebo group compared to probiotic group, as we see in Table 3 below.

**Table 3:** The effect of probiotic on delta SCFA metabolite

**Characteristic SCFA(mM)**	**Total respondent**				

	**Placebo Group**		**Probiotic Group**		

	**Increasing**	**Decreasing**	**Increasing**	**Decreasing**	p-value*
Acetate	31%	57%	69%	43%	0.117
Propionate	37%	52%	63%	48%	0.328
Butyrate	31%	57%	69%	43%	0.117

* Chi Square test

## DISCUSSION

Numbers of studies had been evaluated to the relationship between age, weight and constipation prevalence, one of them is colonic transit time slows down with ageing, although this is highly variable. On the other hand, BMI >25.0 kg/m^2^ positively associated with prevalence of constipation related with low fiber consumption and daily activity (8). Inline with Bellini et al. (2017) that majority of the subjects were women with 49.6 ± 16.6 years old and BMI was 23.7 ± 4.0 kg/m^2^ (9). Aging alone alters the gut microbiota (dysbiosis) that influenced the SCFA metabolite’s that related with fuctional constipation (10, 11). In our experiment, aging showed a negative correlation with SCFA metabolite characteristic, differ with BMI. Based on the mechanism that SCFA influenced defecation process (one of them through motility) so aging and decreasing of BMI inline with the increasing of functional constipation, because decreasing of SCFA will be influenced the motility in the intestinal. The impact of aging on the intestinal barrier and immune system was recently reviewed, that affects the intestinal epithelial barrier and the neural control of smooth muscle contractility. Meerveld et al., (2017) compared colonic permeability via age-associated remodeling of intestinal epithelial tight junction proteins of old baboons to young baboons. They discovered that there is significant tight junction remodeling including a decrease in ZO-1 (Zonula occludens-1), occludin, and JAM-A (Junction Adhesion Molecule-A) proteins and an increase in claudin-2 expression in old baboon colon compared to young, besides that they also found that pro-inflammatory cytokine modulate intestinal permeability through tight junction (12). Tobe et al. (2011) also found that positive effect of SCFAs on flagellar expression on the experiment with enterohemorrhagic *Escherichia coli* (EHEC). Butyrate activated the *flh*DC regulatory genes through leucine-responsive regulatory protein (Lrp). Bacterial motility and the expression of flagella are strictly regulated by a cascade of three transcriptional steps and a response to environmental factors (13). Besides its effects on intestinal epithelial cells, butyrate can also modulate the activity of the enteric nervous system (ENS), increasing the proportion of cholinergic neurons translating to increased gut motility. In contrast to butyrate, propionate seems to decrease colon motility, but increases secretory activity of the colon as well as the number of vasoactive intestinal peptide (VIP) neurons in the intestine (14). According to correlation between BMI and SCFA, Teixeria et al. (2012) also found that preliminary tool for the prediction of abdominal adiposity and metabolic syndrome screening was associated with the increasing of SCFA concentration. Acetate and especially propionate are signalling molecules for the G Protein Coupled Receptor 41 (GPR41) receptor, the activation of which increases host adiposity (15). Fermentation of dietary fiber leads to the production of SCFAs via various biochemical pathways. The size of the letters symbolizes the ratio of SCFAs present. In the distal gut, SCFAs can enter the cells through diffusion or SLC5A8-mediated transport and act as an energy source or an histone deacetylases (HDAC) inhibitor. Luminal acetate or propionate sensed by GPR41 and GPR43 releases Peptide YY (PYY) and Glucagon Like Peptide-1 (GLP-1), affecting satiety and intestinal transit. Luminal butyrate exerts anti-inflammatory effects via GPR109A and HDAC inhibition, SCFAs can also act on other sites in the gut, like the ENS, where they stimulate motility and secretory activity (14). Acetate, propionate and butyrate has a beneficial effect in gastrointestinal tract. Butyrate was a key promoter of colonic health and the main provider of energy for the colonocytes also inhibits IL-12 and increases IL-10 production. Propionate is a potent activator of GPR43 as a receptor SCFA, that is present in immune, nervous and endocrine cells along the entire gastrointestinal tract (16). According to our data, Acetate baseline and end line data was higher than propionate and butyrate (see Table 2 above). Referring to Besten, the affinity of colonocyte relative higher for butyrate, colonocytes prefer butyrate to acetate and propionate, and oxidize it to ketone bodies and CO_2_. According to the Besten and Nourrisson, mostly acetate is predominant in faeces sample, maybe because production pathways of acetate are widely distributed among bacterial groups where as pathways for propionate, butyrate and lactate production appear more highly conserved and substrate specific, this finding was inline with our data that we found acetate was higher than propionate and butyrate (17, 18).

We explored the SCFA profile of Indonesian women with functional constipation before and after probiotic supplementation, longitudinally in the setting of improvement parameter of constipation symptom based on Rome IV criteria. Referring to the Chassard and Farup study, we found that SCFA titers (butyrate, propionate and acetate) in Indonesian women with functional constipation were different (higher) than western and Asian people. Accurate experiment measurement SCFA production *in vivo* is difficult and variative, caused by strong variation of the microbial population between individuals (16, 19–22).

Short chain fatty acids (SCFA) are produced as metabolites in the colon, which mostly colonized with bacteria known as saccharolytic intestinal bacteria. Indigestible of carbohydrates and proteins are not absorbed in the small intestinal tract during the digestion process and will be fermented by commensal microbiota in colon (23) and absorbed in the colon (large intestine) and affected by food intake. SCFA had physiological effect to the ecological environment of the digestive tract (intestine), affect the physiology of the colon, the energy source of host cells and microbiota, the mechanism of the signaling system in the human body (24).

Between SCFA, butyric acid is short chain fatty acid that act as the main energy for colonocytes and regulated immune system also factor that stimulates the growth and differentiation of colonocyte cells. Butyrate is produced in the large intestine (colon), Propionate contributing to gluconeogenesis in the liver, and acetate achieving the highest systemic concentrations in blood. Butyrate and Propionate as these two acids are most often considered to benefit health, including protection against colorectal cancer in the case of butyrate and promotion of satiety and reduction in cholesterol in the case of propionate. About 83% of total SCFA in the mammalian large intestine is acetate, propionic, and butyric acid. This total butyric concentration in the intestinal lumen ranges from 60 mmol/kg to 150 mmol/kg. The amount of faecal SCFA is also relatively constant in the following sequence from a decrease in concentration: acetate> propionate ≥ butyrate. In the colon epithelial cells, butyrate and propionate is completely metabolized; only a small portion enters the bloodstream. (11, 25–27). Sun et al. (2019) observed the alterations in fecal SCFAs in IBS (Irritable Bowel Syndrome) patients, and they found that there were differences between patients with IBS and HCs. In IBS-C patients, propionate and butyrate were reduced, inline with this result, Farub et al. (2016) also found that faecal SCFA could be non-invasive, valid and reliable biomarker for the differentiation of healthy subjects from subjects with IBS, especially Butyrate and Propionate (16, 28). Interestingly, when Gargari et al. (2018) distinguish IBS subtypes based on SCFA’s they concluded that self-perpetuating mechanism in which an initial modified colon transit time, in turn leads to modification of Dysbiosis and SCFA’s metabolite and finally influenced intestinal motility (29).

We have investigated the effects of a probiotic fermented milk containing *Lactobacillus plantarum* IS 10506 10^10^ CFU/day for 21 days in adult women who were diagnosed with functional constipation according to Rome IV criteria. According to the Ibarra et al., combination of *Bifidobacterium animalis* sub-sp. lactis HN019 10^10^ CFU/day for 28 days, increased Bowel Movement Frequency ≤ 3/week and decreased the degree of straining compared with placebo. This activity has been proposed to be attributed to the capacity of probiotics to alter the gatrointestinal microflora, improve intestinal motility, and alter bio-chemical factors (30).

The hierarchy of neural control of gut motility is as follows: the primary regulator of gut motility is Enteris Nervous System (ENS), followed by Autonomic Nervous System (ANS) and then Central Nervous System (CNS). Simultaneously, the immune system, gut secretions, gastrointestinal microbiota, and products of fermentation interact and modulate gut motility via the modulation of afferent sensory nerves that influence gut motility. Serotonin is produced in both the ENS and CNS and is a key neurotransmitter that plays pivotal role in mediating motor and secretory responses in the ENS. Bidirectional “microbiota-gut-brain axis” which has a key role in regulating gut motility. The gastrointestinal microbiota play a vital role in gut motility for example colonization with *L. acidophilus, Bifidobacterium bifidum*, or *Clostridium tabificum* in germfree rats also normalized the small-bowel migrating motor complexes and gut transit time, whereas colonization with *E. coli* inhibited intestinal myoelectric activity (31–33). Interestingly not only probiotic, but also prebiotic like Psyllium husk, derived from the seeds of *Plantago ovate*, after six-day period supplementation Psyllium there was an change in transit time, faecal water content and the concentration of SCFAs, in constipated subject but showed very little change in the healthy subjects (34, 35).

Based on the underlying mechanism that had been decribed before, there is a tendency that probiotic influenced the improvement symptom, SCFA assumed to be factor that influenced mechanism underlyng this effect of probiotic based on the improvement symptom. Butyrate production influenced Constipation-Irritable Bowel Syndrome (C-IBS), act as an anti-inflammatory effects, protect colonic defence barrier and also has a physiology prosess to decrease oxidative stress in the intestine. Butyrate oxidation by colonocytes was further shown to be altered by increasing H_2_S concentration. The functional dysbiosis observed in C-IBS microbiota may have important clinical implications such as abdominal pain, modulation of gut transit and gas related symptoms, due to changes in metabolism output, and plays a major role in balancing and maintaining the physiology in gut. Besides the clinical implication, bile acid deficiency or excess SCFA contribute to the motility in Gut ecosystem and pathophysiology of constipation (4, 19, 34, 36).

Supplementation of *Lactobacillus plantarum* IS 10506 as an indigineous probiotic from West Sumatra Indonesia, proved has a beneficial effect to improve the SCFA metabolite in women with functional constipation. Zhao et al., summarized the mechanism of probiotic influeced the SCFA in constipation people (1):

1. modify the altered intestinal microbiota in patients with constipation;
2. probiotic metabolites may alter gut sensation and motility function
3. probiotics may regulate the intraluminal environment, such as increasing the end products of bacterial fermentation, reducing luminal pH.

## CONCLUSION

After 21 days supplementation, we found that SCFA has a negative correlation with age and positive correlation with BMI. Acetate is the highest SCFA titer found in faeces sample women with functional constipation. Probiotic *Lactobacillus plantarum* IS 10506 supplementation influenced all the SCFA parameter (Acetate, Propionate and Butyrate).

## References

[1] Indonesian Society of Gastroenterology (ISG) National consensus on the management of constipation in indonesia 2010. Acta Med Indones 2011; 43: 267– 274. 22156361

[2] Zhao Y Yu Y-B Intestinal microbiota and chronic constipation. Springerplus 2016; 5: 1130. 27478747 10.1186/s40064-016-2821-1 PMC4951383

[3] Drossman DA Hasler WL Rome IV-Functional GI disorders: Disorders of gut brain interaction. Gastroenterology 2016; 150: 1257– 1261. 27147121 10.1053/j.gastro.2016.03.035

[4] Ivanov II Littman DR Segmented filamentous bacteria take the stage. Mucosal Immunol 2010; 3: 209– 212. 20147894 10.1038/mi.2010.3 PMC3010405

[5] Ewald N ( 2016). Analysis of short chain fatty acids in faecal samples. Swedish University of Agricultural Sciences, Department of Food Science Uppsala. Swedish.

[6] Iebba V Totino V Gagliardi A Santangelo F Cacciotti F Trancassini M Eubiosis and dysbiosis: the two sides of the microbiota. New Microbiol 2016; 39: 1– 12. 26922981

[7] Kim CH Park J Kim M Gut microbiota-derived short-chain fatty acids, T cells, and inflammation. Immune Netw 2014; 14: 277– 288. 25550694 10.4110/in.2014.14.6.277 PMC4275385

[8] Parthasarathy G Chen J Chen X Chia N O’Connor HM Wolf PG Relationship between microbiota of the colonic mucosa vs feces and symptoms, colonic transit, and methane production in female patients with chronic constipation. Gastroenterology 2016: 150: 367– 379.e1. 26460205 10.1053/j.gastro.2015.10.005 PMC4727996

[9] Collado MC Surono I Meriluoto J Salminen S Indigenous dadih lactic acid bacteria: Cell-surface properties and interactions with patogens. J Food Sci 2007; 72: M89– 93. 17995806 10.1111/j.1750-3841.2007.00294.x

[10] Seong-Eun K Colonic slow transit can cause changes in the gut environment observed in the Elderly. J Neurogastroenterol Motil 2017; 23: 3– 4. 28049861 10.5056/jnm16215 PMC5216627

[11] Bellini M Usai-Satta P Bove A Bocchini R Galeazzi F Battaglia E Chronic constipation diagnosis and treatment evaluation: the “CHRO.CO.DI.T.E.” study. BMC Gastroenterol 2017; 17: 11. 28088179 10.1186/s12876-016-0556-7 PMC5237544

[12] Koliada A Syzenko G Moseiko V Budovska L Puchkov K Perederiy V Association between body mass index and Firmicutes / Bacteroidetes ratio in an adult. BMC Microbiol 2017; 17: 120. 28532414 10.1186/s12866-017-1027-1 PMC5440985

[13] Louis P Flint HJ Formation of propionate and butyrate by the human colonic microbiota. Environ Microbiol 2017; 19: 29– 41. 27928878 10.1111/1462-2920.13589

[14] Meerveld BG Johnson AC Grundy D Gastrointestinal Physiology and Function. 2017; Springer International Publishing AG. 10.1007/164_2016_118 28176047

[15] Tobe T Nakanishi N Sugimoto N Activation of motility by sensing short-chain fatty acids via two steps in a flagellar gene regulatory cascade in enterohemorrhagic *Escherichia coli*. Infect Immun 2011; 79: 1016– 1024. 21149585 10.1128/IAI.00927-10 PMC3067497

[16] Koh A Vadder DF Kovatcheva-Datchary P Bäckhed F From dietary fiber to host physiology: short-chain fatty acids as key bacterial metabolites. Cell 2016; 165: 1332– 1345. 27259147 10.1016/j.cell.2016.05.041

[17] Teixeira TF Grześkowiak Ł Franceschini SC Bressan J Ferreira CL Peluzio MC Higher level of faecal SCFA in women correlates with metabolic syndrome risk factors. Br J Nutr 2013: 109: 914– 919. 23200109 10.1017/S0007114512002723

[18] Farup PG Rudi K Hestad K Faecal short-chain fatty acids-a diagnostic bioprofile for irritable bowel syndrome?. BMC Gastroenterol 2016; 16: 51. 27121286 10.1186/s12876-016-0446-z PMC4847229

[19] Besten G Eunen K Groen AK Venema K Reijngoud DJ Bakker BM The role of short-chain fatty acids in the interplay between diet, gut microbiota, and host energy metabolism. J Lipid Res 2013; 54: 2325– 2340. 23821742 10.1194/jlr.R036012 PMC3735932

[20] Venegas DP De la Fuente MK Landskron G González MJ Quera R Dijkstra G Short chain fatty acids (SCFAs)-mediated gut epithelial and immune regulation and its relevance for inflammatory bowel diseases. Front Immunol 2019: 10: 277. 30915065 10.3389/fimmu.2019.00277 PMC6421268

[21] Haenen D Zhang J Souza da Silva C Bosch G van der Meer IM van Arkel J A diet high in resistant starch modulates microbiota composition, SCFA concentrations, and gene expression in pig intestine. J Nutr 2013: 143: 274– 283. 23325922 10.3945/jn.112.169672

[22] Huda-Faujan N Abdulamir AS Fatimah AB Anas OM Shuhaimi M Yazid AM The impact of the level of the intestinal short chain fatty acids in inflammatory bowel disease patients versus healthy subjects. Open Biochem J 2010; 4: 53– 58. 20563285 10.2174/1874091X01004010053 PMC2887640

[23] Nourrisson C Scanzi J Pereira B NkoudMongo C Wawrzyniak I Cian A Blastocystis is associated with decrease of fecal microbiota protective bacteria: comparative analysis between patients with irritable bowel syndrome and control subjects. PLoS One 2014; 9( 11): e111868. 25365580 10.1371/journal.pone.0111868 PMC4218853

[24] Chassard C Dapoigny M Scott KP Crouzet L Del’homme C Marquet P Functional disbiosis within the gut microbiota of patients with constipated-irritable bowel syndrome. Aliment Pharmacol Ther 2012; 35: 828– 838. 22315951 10.1111/j.1365-2036.2012.05007.x

[25] Quigley EMM Spiller RC Constipation and the microbiome: Lumen Versus Mucosa! Gastroenterology 2016; 150: 300– 303. 26710987 10.1053/j.gastro.2015.12.023

[26] Ríos-Covián D Ruas-Madiedo P Margolle A Gueimonde M De los Reyes-Gavilán CG Salazar N Intestinal short chain fatty acids and their link with diet and human health. Front Microbiol 2016; 7: 185. 26925050 10.3389/fmicb.2016.00185 PMC4756104

[27] Esquivel-Elizondo S Ilhan ZE Garcia-Peña EI Krajmalnik-Brown R Insights into butyrate production in a controlled fermentation system via gene predictions. mSystems 2017; 2( 4): e00051– 17. 28761933 10.1128/mSystems.00051-17 PMC5516221

[28] Hamer HM Jonkers D Venema K Vanhoutvin S Troost FJ Brummer RJ Review article: the role of butyrate on colonic function. Aliment Pharmacol Ther 2008; 27: 104– 119. 17973645 10.1111/j.1365-2036.2007.03562.x

[29] Wang L Zhang J Guo Z Kwok L Ma C Zhang W Effect of oral consumption of probiotic *Lactobacillus planatarum* P-8 on fecal microbiota, SIgA, SCFAs, and TBAs of adults of different ages. Nutrition 2014; 30: 776– 783.e1. 24984992 10.1016/j.nut.2013.11.018

[30] Sun Q Jia Q Song L Duan L Alterations in fecal short-chain fatty acids in patients with irritable bowel syndrome: A systematic review and meta-analysis. Medicine (Baltimore) 2019; 98( 7): e14513. 30762787 10.1097/MD.0000000000014513 PMC6408019

[31] Gargari G Taverniti V Gardana C Cremon C Canducci F Pagano I Fecal Clostridiales distribution and short-chain fatty acids reflect bowel habits in irritable bowel syndrome. Environ Microbiol 2018: 20: 3201– 3213. 29749705 10.1111/1462-2920.14271

[32] Ibarra A Latreille-Barbierb M Donazzolob Y Pelletierb X Ouwehand AC Effects of 28-day Bifidobacterium animalis subsp. lactis HN019 supplementation on colonic transit time and gastrointestinal symptoms in adults with functional constipation: A double-blind, randomized, placebo-controlled, and dose-ranging trial. Gut Microbes 2018; 9: 236– 251. 29227175 10.1080/19490976.2017.1412908 PMC6219592

[33] Husebye E Hellstrom PM Sundler F Chen J Midtvedt T Influence of microbial species on small intestinal myoelectric activity and transit in germ-free rats. Am J Physiol Gastrointest Liver Physiol 2001; 280: G368– 380. 11171619 10.1152/ajpgi.2001.280.3.G368

[34] Bercik P Collins SM Verdu EF Microbes and the gutbrain axis. Neurogastroenterol Motil 2012: 24: 405– 413. 22404222 10.1111/j.1365-2982.2012.01906.x

[35] Dimidi E Christodoulides S Scott M Whelan K Mechanisms of action of probiotics and the gastrointestinal microbiota on gut motility and constipation. Adv Nutr 2017: 8: 484– 494. 28507013 10.3945/an.116.014407 PMC5421123

[36] Jalanka J Major G Murray K Singh G Nowak A Kurtz C The effect of psyllium husk on intestinal microbiota in constipated patients and healthy controls. Int J Mol Sci 2019: 20: E433. 30669509 10.3390/ijms20020433 PMC6358997

